# Transcriptome analysis of complex I-deficient patients reveals distinct expression programs for subunits and assembly factors of the oxidative phosphorylation system

**DOI:** 10.1186/s12864-015-1883-8

**Published:** 2015-09-15

**Authors:** Robin van der Lee, Radek Szklarczyk, Jan Smeitink, Hubert J M Smeets, Martijn A. Huynen, Rutger Vogel

**Affiliations:** 1 Centre for Molecular and Biomolecular Informatics, Radboud Institute for Molecular Life Sciences, Radboud university medical center, PO BOX 9101, 6500 HB Nijmegen, The Netherlands; 2 Department of Clinical Genetics, Unit Clinical Genomics, Maastricht University Medical Centre, 6200 MD Maastricht, The Netherlands; 3 Nijmegen Center for Mitochondrial Disorders, Department of Pediatrics, Radboud Institute for Molecular Life Sciences, Radboud university medical center, PO BOX 9101, 6500 HB Nijmegen, The Netherlands; 4 Unit Clinical Genomics, Department of Genetics and Cell Biology, School for Growth and Development and for Cardiovascular Research, Maastricht University Medical Centre, Maastricht, The Netherlands

**Keywords:** Mitochondria, Mitochondrial disorders, Oxidative phosphorylation, Complex I, Assembly factors, Biogenesis, Gene expression, Co-expression, Transcription regulation, Transcription factors

## Abstract

**Background:**

Transcriptional control of mitochondrial metabolism is essential for cellular function. A better understanding of this process will aid the elucidation of mitochondrial disorders, in particular of the many genetically unsolved cases of oxidative phosphorylation (OXPHOS) deficiency. Yet, to date only few studies have investigated nuclear gene regulation in the context of OXPHOS deficiency. In this study we performed RNA sequencing of two control and two complex I-deficient patient cell lines cultured in the presence of compounds that perturb mitochondrial metabolism: chloramphenicol, AICAR, or resveratrol. We combined this with a comprehensive analysis of mitochondrial and nuclear gene expression patterns, co-expression calculations and transcription factor binding sites.

**Results:**

Our analyses show that subsets of mitochondrial OXPHOS genes respond opposingly to chloramphenicol and AICAR, whereas the response of nuclear OXPHOS genes is less consistent between cell lines and treatments. Across all samples nuclear OXPHOS genes have a significantly higher co-expression with each other than with other genes, including those encoding mitochondrial proteins. We found no evidence for complex-specific mRNA expression regulation: subunits of different OXPHOS complexes are similarly (co-)expressed and regulated by a common set of transcription factors. However, we did observe significant differences between the expression of nuclear genes for OXPHOS subunits versus assembly factors, suggesting divergent transcription programs. Furthermore, complex I co-expression calculations identified 684 genes with a likely role in OXPHOS biogenesis and function. Analysis of evolutionarily conserved transcription factor binding sites in the promoters of these genes revealed almost all known OXPHOS regulators (including GABP, NRF1/2, SP1, YY1, E-box factors) and a set of novel candidates (ELK1, KLF7, SP4, EHF, ZNF143, and TEL2).

**Conclusions:**

OXPHOS genes share an expression program distinct from other genes encoding mitochondrial proteins, indicative of targeted nuclear regulation of a mitochondrial sub-process. Within the subset of OXPHOS genes we established a difference in expression between mitochondrial and nuclear genes, and between nuclear genes encoding subunits and assembly factors. Most transcription regulators of genes that co-express with complex I are well-established factors for OXPHOS biogenesis. For the remaining six factors we here suggest for the first time a link with transcription regulation in OXPHOS deficiency.

**Electronic supplementary material:**

The online version of this article (doi:10.1186/s12864-015-1883-8) contains supplementary material, which is available to authorized users.

## Background

Mitochondria are the primary source of cellular ATP, which is generated via electron transfer in the oxidative phosphorylation (OXPHOS) system using substrates derived from oxidation of carbohydrates, fatty acids and amino acids. The value of healthy mitochondria becomes evident in cases of OXPHOS deficiency. These metabolic disorders primarily affect tissues with a high ATP demand such as the brain, heart, and skeletal muscle, typically resulting in progressive energy deficiencies and childhood death [1]. Respiratory chain disorders occur in approximately 1:5,000–10,000 living births [2]. The most frequently encountered one is complex I deficiency (OMIM: 252010). No cure for OXPHOS deficiencies exists, and current interventions are either cumbersome or only effective for specific types of the disease [3]. Furthermore, 40–70 % of cases remain genetically unexplained [4, 5] as no mutations are found in the genes encoding structural subunits or assembly factors, impeding genetic counseling. Therefore there is a great need for a better understanding of how the biogenesis and activity of the OXPHOS system is controlled.

Cells can control metabolic output by regulating gene expression. The OXPHOS system is constructed from a combination of nuclear and mitochondrial gene products, e.g. seven genes of complex I are encoded by the mitochondrial DNA and 37 by the nuclear DNA. This bigenomic assembly implies that there are at least two mechanisms for regulating OXPHOS gene expression: mitochondrial and nuclear. Replication, maintenance, and transcription of mitochondrial DNA are tightly regulated processes. Disturbances in any of these processes have been firmly linked to combined OXPHOS deficiency (for a recent review see [6]). In contrast, although much has been published about the relevance of metabolic (co)-regulators such as PGC-1α, NRF1, NRF2, YY1, and SP1 for the regulation of OXPHOS gene expression, little is known about the possible relationship between disturbed nuclear gene regulation and OXPHOS deficiency.

In this study, we investigate mitochondrial and nuclear gene expression patterns in patients with complex I deficiency under various conditions of perturbed mitochondrial metabolism. Gene expression clustering, co-expression calculations and analysis of transcription factor binding sites provide insights into nuclear transcription regulation of OXPHOS, suggesting regulation of the system as a whole rather than regulation of specific complexes. Our data also reveal that assembly factors follow an expression pattern that is more like genes encoding other mitochondrial proteins than like OXPHOS subunits. Finally, analysis of enriched regulators of nuclear genes co-expressing with complex I not only retrieves virtually all transcription factors (TFs) with a well-known role in the regulation of OXPHOS gene expression, but also identifies several factors not previously implicated in the regulation of OXPHOS in general or in respiratory chain disease.

## Results

### Culturing, incubation and RNA sequencing of complex I-deficient patient cells

**Fig. 1:**
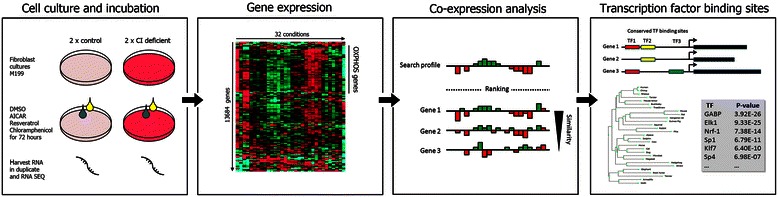
Overview of the approach. Two control and two complex I-deficient patient fibroblast cell lines were incubated for three days with vehicle (DMSO) and three compounds that trigger a metabolic response and RNA was harvested in duplicate for RNA sequencing. Expression values of individual genes across the resulting 4 × 4 × 2 = 32 samples were measured, normalized and clustered. This allowed for the analysis of expression profiles per gene. Genes were ranked based on the similarity of their expression profile (co-expression) with the average profile for a bait set of genes, such as the OXPHOS system or complex I. High ranking genes were analyzed for the presence of conserved transcription factor binding sites across 29 mammals. Common, over-represented binding sites are (potential) transcriptional regulators of the system

To investigate patterns of transcription in OXPHOS deficiency, we measured gene expression in two healthy fibroblast cell lines and two fibroblast patient cell lines carrying mutations in complex I genes NDUFS2 and ND5 (Fig. 1). Cells were treated with vehicle (DMSO) and three compounds that stimulate or inhibit mitochondrial metabolism in order to trigger a transcriptional response of mitochondrial genes. Chloramphenicol inhibits mitochondrial gene translation and OXPHOS assembly and function [7]. Resveratrol stimulates mitochondrial growth/metabolism via SIRT1/AMPK [8]. AICAR stimulates mitochondrial metabolism as an AMPK agonist [9]. The compound incubations were done in duplicate, resulting in a total of 4 cell lines × 4 compounds × 2 replicates = 32 samples. Processing of the RNA sequencing data revealed expression values of 13,684 nuclear DNA encoded genes (referred to as nuclear genes) and 16 mitochondrial DNA encoded genes (referred to as mitochondrial genes) in all samples. We subsequently analyzed the transcriptomes using three approaches: gene expression clustering, co-expression calculations, and transcription factor binding site analysis (Fig. 1).

Of our list of currently known 127 unique OXPHOS subunits and assembly factors (Additional file 1: Table S1), all 13 mitochondrial and 112 nuclear OXPHOS genes were detected in the expression analysis. To investigate the distribution of OXPHOS genes in the 13,700 expression profiles we performed hierarchical clustering (Pearson uncentered, average linkage). Many OXPHOS genes share a distinct expression profile across the experiments and are significantly enriched among a sub-cluster of 1,518 genes (48/1,518 were OXPHOS, Fisher’s exact *P* < 0.05, Additional file 2: Figure S1 and Additional file 3: Table S2). Next we analyzed mitochondrial and nuclear OXPHOS gene expression separately.

### Differences in expression of mitochondrial OXPHOS genes between cell lines and treatments

mRNA measurements for mtDNA-encoded genes are strongly correlated between biological replicates (R^2^ = 0.88, see Methods). Total mitochondrial mRNA expression was not significantly different between the four cell lines (one-way ANOVA *P =* 0.6) indicating that differences in the genetic background of our cells do not have a systematic influence on total mitochondrial gene expression. mRNA expression of mitochondrial genes in control vs. patient cells differed less than 1 % implying that the complex I defects do not influence global mtDNA expression levels **(**Additional file 2: Figure S2A**)**. We did, however, observe a significant transcriptional response in cells upon perturbation of metabolism using different compounds (4 treatments, one-way ANOVA *P =* 0.0002). The total mitochondrial mRNA expression was 15 % higher upon chloramphenicol treatment compared to control DMSO, and significantly higher than any of the other treatments (P < 0.0003 in pairwise comparison with the other groups, two-tailed paired *T*-test) (Additional file 2: Figure S3). We observed high transcript abundance upon chloramphenicol treatments in all cell types. The variance in chloramphenicol-induced gene expression is very low compared to other treatments and the control condition, suggesting saturation of mitochondrial transcript abundance (Additional file 2: Figure S3). Total mtDNA gene expression levels in AICAR and resveratrol treatments are lower than control 4 % (*P =* 0.1) and 5 % (*P =* 0.001), respectively.

**Fig. 2:**
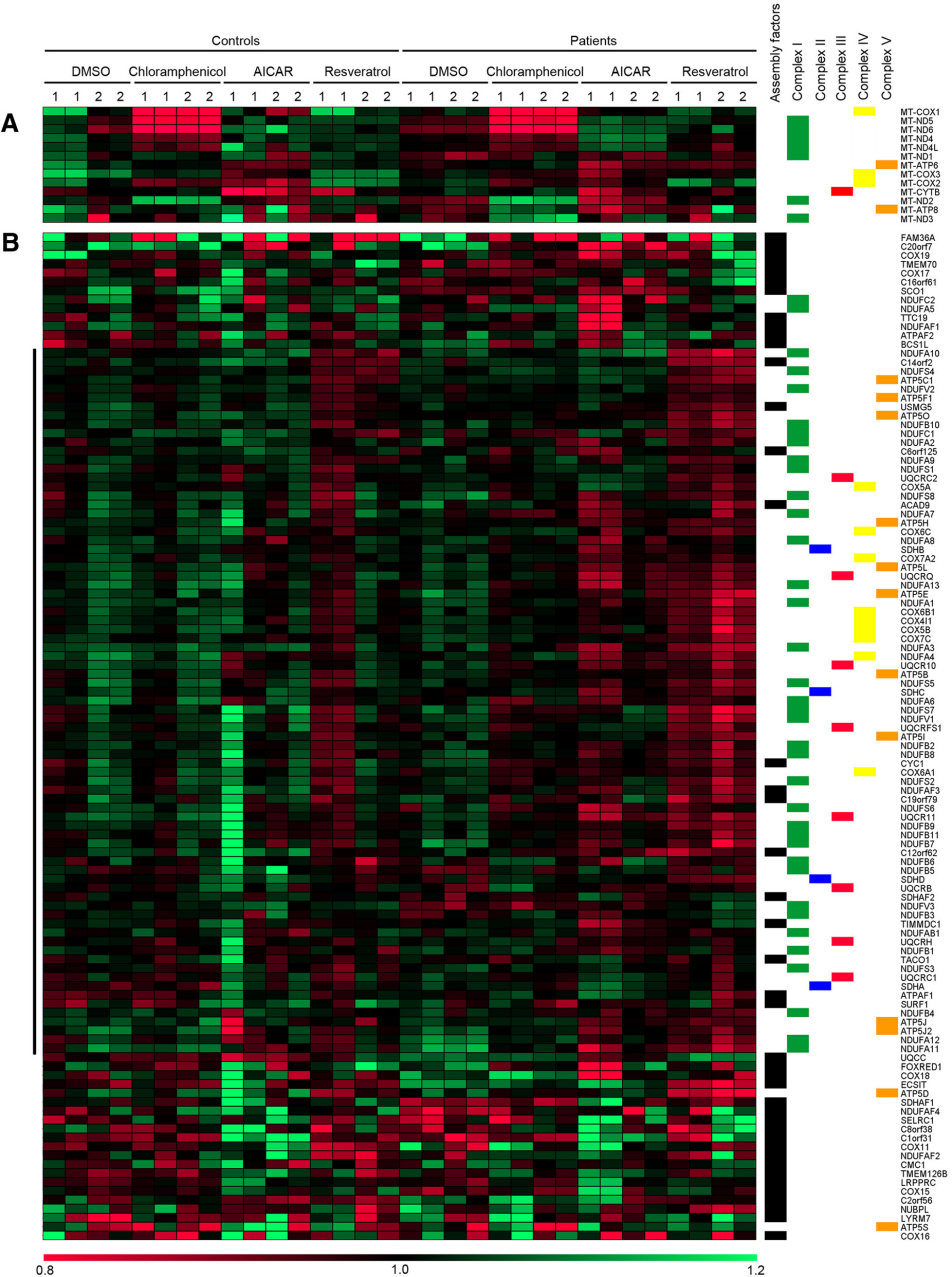
Mitochondrial OXPHOS genes respond differentially to treatments and assembly factors tend to express differently from nuclear genes encoding OXPHOS subunits. Expression profiles of OXPHOS genes are shown in heatmap representation in 32 RNA sequencing measurements of control and complex I-deficient patient cells. Panel **a** shows the mitochondrial-encoded OXPHOS genes. Panel **b** shows the nuclear-encoded OXPHOS genes. At the top of the figure, controls, patients, and compound incubations are indicated across the samples, where numbers 1 and 2 refer to the cell line. On the right assembly factors and subunits per complex are labeled by color. On the left the central cluster of OXPHOS subunit genes is indicated by a vertical bar. Genes were clustered using average linkage clustering with uncentered Pearson correlation as distance matrix. On the bottom the horizontal bar depicts expression values. A value of 1.0 (black) denotes median log-expression of the gene (see Methods), with green denoting higher and red denoting lower expression levels

mRNA levels of mitochondrial OXPHOS genes change in response to treatments (Fig. 2a). These changes in transcript levels can be the result of a combination of factors, such as changes in mitochondrial transcription, in mitochondrial mRNA degradation rates, and in trafficking. The direction of mRNA changes upon treatments vary considerably between genes even within the same complex. For example, the transcript level of MT-ND2 increases by 50 % upon chloramphenicol treatment compared to vehicle, while other subunits of complex I (MT-ND4, MT-ND5, MT-ND6) decrease up to 30 % (Fig. 2a and Additional file 2: Figure S4**)**. We did not observe correlations between changes in mitochondrial gene expression and respiratory chain complex, position on the mitochondrial genome or mitochondrial strand of origin (data not shown). However, certain trends are apparent and reproducible. Up-regulation of genes encoding complex I subunits: MT-ND4, 4L, 5 and 6 upon AICAR treatment (~40 %) and their down regulation in chloramphenicol (~10 %) is significant compared to cells treated with control DMSO (P < 0.05 or less for these genes, two-tailed paired *T*-test). MT-ND2 and, to a lesser extent MT-ND3 and MT-ATP8 show the opposite expression pattern: they are up regulated in chloramphenicol compared to AICAR (140–270 %, *P <* 0.05). Thus, mitochondrial gene expression is similar between cell lines but different upon treatment versus control vehicle, as subsets of mitochondrial OXPHOS genes respond differently to chloramphenicol and AICAR.

### Differences in expression of nuclear OXPHOS genes between cell lines and treatments

Examination of the expression data for nuclear genes suggests differences in steady state and treatment-induced OXPHOS mRNA levels between control and patient cell lines, with control 2 generally showing higher nuclear OXPHOS gene expression than the other cell lines (Additional file 2: Figure S2B). Furthermore, the chloramphenicol-induced accumulation of transcripts for a subset of mitochondrial OXPHOS genes (Fig. 2a) is not matched by nuclear OXPHOS mRNA levels (Fig. 2b). For nuclear complex I genes, unsupervised clustering captures similarity of transcriptional responses to AICAR and resveratrol in closely-knit clusters (Additional file 2: Figure S6), but these responses are not consistent for all genes. Nevertheless, combining the gene expression profiles across all cell lines and treatment conditions is highly informative, as will be illustrated in the following sections.

### Clustering identifies distinct expression patterns of OXPHOS subunits and assembly factors

Detailed analysis of the expression levels of nuclear OXPHOS genes revealed no specific clusters for individual OXPHOS complexes, arguing against complex-specific regulation of expression for the evaluated cell types and conditions (Fig. 2b). However, clustering did highlight a distinction between the expression profiles of OXPHOS subunits and assembly factor genes: 67 of the 81 genes (83 %) in the central largest cluster are subunit genes, while 28 of the 32 genes (82 %) in the remaining smaller clusters are assembly genes (Fig. 2b). Thus, although OXPHOS genes share similar expression profiles, subunits cluster together in a distinct group from OXPHOS assembly genes, indicative of differential transcription regulation.

### Co-expression confirms absence of OXPHOS complex-specific expression profiles and differential behavior of assembly factors

**Fig. 3:**
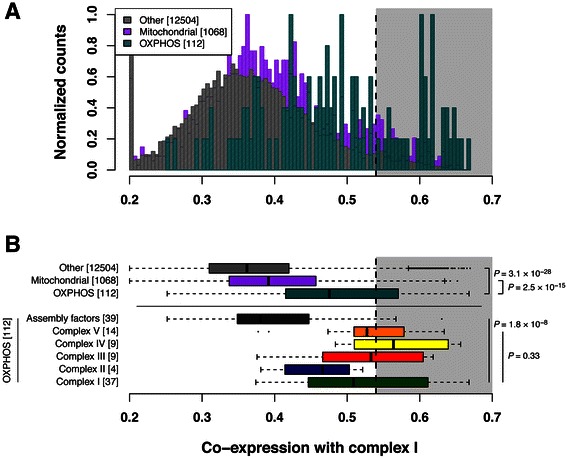
All OXPHOS complexes co-express with complex I, but assembly factors follow divergent transcriptional programs. Histograms (**a**) and boxplots (**b**) of co-expression scores with known complex I genes. Genes are grouped as OXPHOS, mitochondrial (nuclear genes encoding proteins with a function in the mitochondria), or other genes in the genome (**a**). OXPHOS genes are further sub-classified into individual complexes and assembly factors (**b**). The shaded grey area represents the top 5 % of nuclear genes co-expressing with complex I, which are included in the TF binding site enrichment analysis. The dashed line marks the cutoff score (0.54). Groups are mutually exclusive, i.e. genes occur only in one group; complex I-V genes together with assembly factors make up the OXPHOS group. Histogram counts (**a**) were normalized to a maximum of 1 for each gene set. Colored boxes in boxplots (**b**) represent the 50 % of data points above (×0.75) and below (×0.25) the median (×0.50; the black line within the box). Vertical lines (whiskers) connected to the boxes by the horizontal dashed lines represent the largest and the smallest non-outlier data points (which are plotted as individual dots). *P* values comparing two groups are from Mann–Whitney *U* tests and those comparing multiple groups are from Kruskal-Wallis tests

To identify additional candidates with expression profiles compatible with OXPHOS genes, we calculated co-expression with complex I for all measured nuclear genes by integrating the gene expression profiles (Fig. 1). As expected, known OXPHOS genes as a group have significantly higher co-expression with complex I than do other genes in the genome (Figs. 3a and b, *P* = 3.1 × 10^−28^, one-tailed Mann–Whitney *U* test). However, OXPHOS genes are also more co-expressed with complex I than other genes encoding proteins localized to the mitochondria [10] (*P* = 2.5 × 10^−15^). Sub-classification of OXPHOS genes into assembly factors and the structural subunits of the five complexes (Additional file 1: Table S1) revealed no complex-specific co-expression patterns, in agreement with the trends observed in the clustering approach above: complex I co-expression distributions are similar for individual OXPHOS complexes (Fig. 3b, *P* = 0.33, Kruskal-Wallis test). In fact, expression profiles of complexes III, IV and V tend to be more similar to the average profile of complex I (i.e. have higher median complex I co-expression scores) than those of complex I genes themselves, supporting the notion that mRNA expression of OXPHOS genes is jointly regulated. The possible exception is complex II, subunits of which tend to show less co-expression with complex I genes (though not statistically significantly different) than do subunits of complexes III, IV and V.

Interestingly, assembly factors (including those involved in assembly of complex I itself) have a significantly lower co-expression with complex I than structural subunits (*P* = 1.8 × 10^−8^, Kruskal-Wallis test, Fig. 3b) and are more similar to other mitochondrial genes, confirming that their expression profiles are significantly different from OXPHOS subunits (Fig. 2b). Thus, the complex I-like expression profile seems specific for OXPHOS subunits compared to assembly factors or other genes encoding mitochondrial proteins, but does not distinguish individual complexes.

Genes that consistently co-express with complex I under different conditions in different cells are likely to be functionally related, for example by being involved in OXPHOS biogenesis or its regulation. To identify such potential genes we considered the top 5 % of complex I co-expression values, corresponding to a co-expression score cutoff of 0.54 (close to a local peak in the frequency distribution of co-expression scores, Figs. 3 and Additional file 2: Figure S5, Additional file 4: Table S3). This cutoff captures a total of 684 nuclear genes, including 43 % (16/37) of the complex I subunits, 42 % (15/38) of the other OXPHOS subunits, three assembly factors (NDUFAF3, ACAD9, C19orf79) and 96 other known mitochondrial genes. Functional classification of these top-ranking genes using DAVID [11] reveals a program that fits the biogenesis and breakdown of OXPHOS proteins (Additional file 5: Table S4). Gene groups with the highest enrichment scores (ES) other than OXPHOS (37 genes, ES 12, mostly complex I/III/IV/V genes) are translation (51 genes, ES 23, mostly (mito)ribosomal genes), ribonuclear processing (12 genes, ES 8) and quality control (14/15 genes, ES 8/6, mostly ubiquitin and proteasome-related genes). That plasticity in transcription of metabolic/mitochondrial genes is tightly coupled to proteolytic breakdown of the involved activators was recently highlighted by Catic et al. [12] and could explain the enrichment of the latter group. Two additional enriched gene groups are of potential interest. The first group (ES 21) contains PRELID1 (mitochondrial morphology and function) [13], CHCHD2 (regulator of cytochrome oxidase) [14], ISOC2 (tumor development) [15], and MRPL53 (a mitochondrial ribosomal protein also found in [16] in a putative ribonucleotide complex with LRPPRC and SLIRP). The second (ES 7) contains 7 genes including the complex I assembly factor ACAD9, ETFA and ETFB (involved in beta oxidation of fatty acids), and C1QBP (mitochondrial protein synthesis) [17].

### Nuclear OXPHOS genes as a group are regulated by a common set of known and novel candidate OXPHOS transcription factors

Next, we analyzed potential transcriptional regulators of the 684 (top 5 %) nuclear genes having the highest co-expression with complex I. We obtained conserved transcription factor binding sites (TFBS) for 218 unique TFs in the promoter regions of 16,298 human genes from a comparative evolutionary analysis of the genomes of 29 placental mammals [18]. This information is independent of experimental conditions and cell type and is therefore typically well suited for exploratory analysis aimed at prioritizing which TFs may regulate a biological system (see Discussion). The data include at least one conserved TFBS in 606 of the 684 genes (89 %) with the highest complex I co-expression values (Fig. 1).

Genes that co-express with complex I are significantly enriched for 12 TFs compared to the background of all human genes after correction for testing multiple TF (Benjamini-Hochberg-corrected Fisher’s exact *P* < 0.05, Table 1 and Additional file 6: Table S5): GABP, Elk1, Nrf-1, Sp1, Klf7, Sp4, Ehf, znf143, Myc, YY1, Ebox, and Tel2. Ten additional TFs are significantly enriched when they are evaluated individually (i.e. without correcting for multiple testing; Fisher’s exact *P* < 0.05, Table 1 and Additional file 6: Table S5).

**Table 1:** Transcription factor binding site enrichment in promoter regions of the top 5 % complex I co-expressing nuclear genes

	

Background: all human genes. Listed are all individually significantly enriched TFs (i.e. without correcting for multiple testing; Fisher’s exact *P <* 0.05). TFs with Benjamini-Hochberg-corrected Fisher’s exact *P* < 0.05 are shaded in grey. TFs with a known role in OXPHOS biogenesis are in bold See Additional file 6: Table S5 for detailed results

Virtually all known OXPHOS transcription regulators are present among these 22 enriched factors, including GABP, NRF1/2, SP1, YY1, E-box. For example, the nuclear respiratory factors 1 and 2 (NRF1 and NRF2) have ranks 3 and 20 (out of 218 TFs tested, *P* = 1.0 × 10^−15^ and 0.033, respectively). Furthermore, NRF1 ranks first in the analysis of known complex I genes, and is also strongly over-represented in complex II-V genes and OXPHOS assembly factors (Table 2). The most significantly enriched TF for complex I co-expressing genes is GABP/NRF2 (35 % of co-expressing genes, 2.1-fold enrichment compared to all genes, *P* = 1.8 × 10^−28^). NRF2 belongs to the ETS family of transcription factors, which also contains ETS1 (rank 17, *P* = 0.013). Other examples of known OXPHOS regulators of complex I co-expressing genes are SP1 (rank 4, *P* = 1.2 × 10^−12^), YY1 (rank 10, *P* = 6.3 × 10^−4^), CREB (rank 21, *P* = 0.041), the E-box regulatory motif (rank 11, *P* = 1.3 × 10^−3^) and E-box factor c-Myc (rank 9, *P* = 2.7 × 10^−5^). PGC-1-related coactivators (PRC), such as PGC-1(α) and PGC-1β, are known to act through PPAR(α) (rank 14, *P* = 8.6 × 10^−3^) and PPARγ (rank 16, *P* = 0.012). Aside from known regulators, our analysis also identified six TFs not previously linked to OXPHOS gene expression (see Discussion): ELK1 (rank 2, *P* = 8.6 × 10^−27^), KLF7 (rank 5, *P* = 1.5 × 10^−11^), SP4 (rank 6, *P* = 1.9 × 10^−8^), EHF (rank 7, *P* = 2.2 × 10^−8^), ZNF143 (rank 8, *P* = 3.6 × 10^−7^), and TEL2 (rank 12, *P* = 2.6 × 10^−3^).

**Table 2:** Transcription factor binding site enrichment in promoter regions of OXPHOS subunits and assembly factors

	

Background: all human genes. Listed are all individually significantly enriched TFs (i.e. without correcting for multiple testing; Fisher’s exact *P <* 0.05). TFs with Benjamini-Hochberg-corrected Fisher’s exact *P* < 0.05 are shaded in grey. Numbers in parentheses indicate the number of genes for which there is conserved TFBS data. Nrf-1 is marked in bold

As we have shown above, expression clustering and co-expression analysis indicate joint regulation of OXPHOS genes as a group, rather than specific regulation of individual complexes. We next asked whether there is also no evidence for complex-specific regulation in the conserved TF binding sites. Promoter regions of complex I subunits are significantly enriched only for Nrf-1 binding motifs (40.6 % of complex I genes, 3.2-fold enrichment, Fisher’s exact *P* = 8.3 × 10^−5^, Table 2A). However, Nrf-1 is not specific for complex I genes as it is also over-represented in subunits of complexes II, III, IV and V (32.4 % of complex II-V genes, 2.5-fold enrichment, Fisher’s exact *P* = 2.6 × 10^−3^, Table 2B) and in OXPHOS assembly factors (25.7 % of assembly factors, 2.0-fold enrichment, Fisher’s exact *P* = 0.030, Table 2C). Thus, it appears that there are no significantly over-represented transcription factors among the 218 tested that regulate specific OXPHOS complexes, confirming the patterns of general OXPHOS subunit regulation observed in the (co-)expression data.

## Discussion

Disorders of the oxidative phosphorylation (OXPHOS) system are rare but devastating energy deficiencies. To date, the genetic basis of a large fraction (estimates range from 40–70 %) of these disorders remains enigmatic [4, 5]. When no mutations are found in any of the known OXPHOS subunits and assembly factors, a possible explanation may be found in the genes that control their expression, such as transcriptional (co-)activators. A specification of which of these factors control OXPHOS gene expression, and how, would be helpful.

Large-scale gene expression analyses have previously revealed co-expression of genes involved in the OXPHOS system [19–25]. The associated transcription program is moderated by a set of (co-)activators, including PGC-1α, NRF1/2, YY1, and SP1. To our knowledge only one study focused on the possibility of individual expression programs for individual OXPHOS complexes [23]. In this study, only genes within OXPHOS complexes I and IV showed moderately higher degrees of co-expression with each other than with OXPHOS genes as a whole. However, no specific TFs (among 150 families tested), conserved across three organisms (human, mouse, rat), could be identified to explain this result.

We aimed to further investigate the existence of separate expression programs for individual OXPHOS complexes. To this end we investigated the transcription program for OXPHOS genes in complex I-deficient cells and assessed the regulatory elements involved. We used complex I deficient cell lines and controls in order to discriminate complex I-related expression responses upon drug treatment. To elicit a metabolic transcriptional response we incubated the cells with and without chloramphenicol, AICAR, and resveratrol. The effects of these incubations were generally similar between controls and patients. Chloramphenicol resulted in an accumulation of mitochondrial mRNA, likely due to the block in translation. Furthermore, after chloramphenicol and AICAR treatment, we observed contrasting changes in the expression of subsets of mitochondrial OXPHOS genes (COX1/ND4/ND4L/ND5/ND6 vs ATP8/ND2/ND3) (Fig. 2a).

Chloramphenicol inhibits mitochondrial translation, hence the observed changes in transcript levels are likely the consequence of disrupted mitochondrial translation. Recent studies have highlighted feedback mechanisms between mitochondrial translation and transcription. For example, ribosome subunit MRPL12 interacts with mitochondrial polymerase POLRMT to enhance mitochondrial transcription [26, 27]. In addition, POLRMT interacts with 12S rRNA methyltransferase h-mtTFB1 as a possible checkpoint for 28S and 55S ribosome assembly [28]. The changes in transcript levels that we observe upon inhibition of translation are not correlated with respiratory chain complex, position on the mitochondrial genome or mitochondrial strand of origin. How the abovementioned interactions could affect the levels of individual mitochondrial transcripts is unclear and likely partly controlled by regulatory proteins. A recent example of such a protein is FASTKD5, required for the maturation of a subset of mitochondrial OXPHOS mRNA’s, primarily COX1 [29].

Although our analysis of mitochondrial and nuclear OXPHOS gene transcription did not reveal complex-specific expression patterns, we did observe a significantly different expression profile for nuclear OXPHOS subunit genes versus other genes encoding proteins localized to the mitochondria, supporting differential nuclear gene regulation of a sub-mitochondrial process. Interestingly, OXPHOS assembly factors showed expression profiles that are significantly different from OXPHOS subunits: assembly factor expression tends to be more similar to non-OXPHOS mitochondrial genes. For example, the iron-sulfur cluster protein NUBPL (IND1) [30] is a complex I assembly factor with an expression profile very different from OXPHOS subunits (Fig. 2b) and low co-expression with complex I subunits (at ~0.3 is has the third lowest co-expression score of all 39 analyzed OXPHOS assembly factors). These findings suggest that expression of at least some assembly factors is controlled by other factors than that of subunits. This is perhaps not surprising considering that assembly factors can play multiple roles not exclusive to the biogenesis of OXPHOS complexes, for example in translation, membrane insertion, or the incorporation of prosthetic groups.

In our expression data of complex I-deficient cells, 684 genes represent the top 5 % of nuclear genes that co-express with known complex I genes. Among these genes are many subunits of other OXPHOS complexes, confirming that different OXPHOS complexes have highly similar expression profiles. Other highly enriched gene groups in the top complex I co-expressing genes are those for translation and for quality control. Of particular interest are a number of genes implicated in RNA processing and a subset of fatty acid oxidation genes. For example, ACAD9 is essential for complex I assembly and plays no obvious role in fatty acid oxidation, despite a highly conserved fatty acid oxidation active site [31, 32]. ACAD9 (Fig. 2b) and two genes actually involved in fatty acid metabolism, ETFA and ETFB, have high co-expression with complex I subunit genes (scores ~0.6), while ACADVL, ACADM, and ACADS, which are evolutionarily related to ACAD9, all have lower scores of ~0.4. Co-expression of ACAD9 and a number of key fatty acid oxidation genes with complex I hints towards a possible functional link between these two metabolic pathways.

To explore which TFs may be important for regulating genes that co-express with complex I, we made use of a previously published data set of TF binding sites that are conserved across 29 mammals [18]. The conserved TFBS are detected solely on the basis of genome sequence and are therefore independent of experimental conditions and cell type [33]. In contrast, binding sites identified in for example ChIP-sequencing experiments, such as generated by the ENCODE consortium [34], are specific to cell type and experimental conditions. Although the conserved TFBS data has been shown to agree well with experimentally measured ChIP-seq binding sites [18], only a subset of TFs have been measured across many different cell types and conditions. Therefore, sequence-conserved TFBS data such as used in this study is typically well suited for prioritizing which TFs may regulate a biological system. Indeed, we also analyzed enrichment of TFs using TFBS data derived from ENCODE ChIP-seq peaks, either by creating various composite data sets that union all tissues and conditions measured, or by analysis of specific cell types relevant to mitochondrial functioning such as skeletal muscle and heart cells. The union data sets produced very large enrichments for almost all TFs tested, while the tissue-specific data lacked power and produced not a single over-represented TF. Thus, neither of these approaches, in our hands, were insightful for prioritizing TFs involved in complex I co-expression, or in fact for various other biological systems generally unrelated to mitochondrial function.

Analysis of conserved TF binding sites in promoter regions revealed 22 over-represented TFs compared with their genomic abundance. The enriched TFs (Table 1) correspond well with known OXPHOS regulators [35, 36]. However, several over-represented TFs have not been previously implicated in the regulation of complex I or OXPHOS in general. For example, ELK1 is the second strongest enriched TF in genes that co-express with complex I (*P* = 8.6 × 10^−27^) and belongs to the ETS family of transcription factors, which also includes known OXPHOS regulators NRF2, GABP, and ETS1. Interestingly, ELK1 has been linked to primary respiratory chain disease: its target genes show large differential expression between muscle cells and fibroblasts of patients [37]. EHF (ESE3, rank 7, *P* = 2.2 × 10^−8^) and TEL2 (ETV7, rank 12, *P* = 2.6 × 10^−3^) are two other ETS family members.

SP4 (rank 6, *P* = 1.9 × 10^−8^), together with SP1 and KLF7 (rank 5, *P* = 1.5 × 10^−11^) part of the Krüppel-like family of TFs, was recently implicated in the regulation of cytochrome c oxidase (OXPHOS complex IV) gene expression in primary neurons [38]. In addition, SP4 regulates the three mitochondrial transcription factors TFAM, TFB1M, and TFB2M, and the complex IV assembly protein SURF1. Thus, the high rank of this TF fits with its proposed role in OXPHOS gene regulation.

Zinc finger protein 143 (ZNF143; complex I co-expression rank 8, *P* = 3.6 × 10^−7^; known complex I genes rank 2, *P* = 7.2 × 10^−3^) is a transcriptional activator for selenocysteine tRNA (tRNAsec). During mitochondrial respiratory chain dysfunction, ZNF143 upregulates tRNAsec, which results in increased expression of glutathione peroxidase 1 (GPX1) [39]. This mechanism has been proposed to protect cells from oxidative stress damage in conditions of respiratory chain dysfunction. In addition, ZNF143 binds to HCFC1, which is a common component of active CpG island promoters and coincides with YY1 and GABP, both relevant to OXPHOS biogenesis [40]. Taken together, ZNF143 is a strongly enriched regulator of genes that co-express with complex I across expression data of complex I-deficient patients. Further investigation of the transcription factors newly identified by our analyses may provide new clues towards gene regulation in deficiencies of the oxidative phosphorylation system.

## Conclusions

To find new leads for explaining the many genetically unexplained cases of OXPHOS deficiency we have explored mitochondrial and nuclear gene expression and transcriptional elements of OXPHOS subunits and assembly factors in human complex I-deficient cells. We found that genes of the OXPHOS system co-express distinctly from other genes encoding mitochondrial proteins but found no support for distinct expression profiles for individual complexes. Genes encoding OXPHOS assembly factors follow an expression program different from that of OXPHOS subunits, suggesting that regulation of biogenesis occurs via different transcriptional activators. Many regulators of genes that co-express with complex I are well-established factors for OXPHOS biogenesis. However, for six factors, we suggest for the first time a link with transcriptional regulation of OXPHOS genes. The physiological relevance of these factors will need to be tested.

## Methods

### Cell culture and RNA isolation

Control (internal culture no #4996 and #MW35) and patient (internal culture no #5170 and #9170) fibroblasts were cultured in M199 medium (Life Technologies) supplemented with 10 % fetal calf serum (v/v) and penicillin/streptomycin. These cultures were exposed for 72 h in the same medium with the addition of 125 uM chloramphenicol, 75 uM resveratrol, 500 uM AICAR, or 0.01 % DMSO (vehicle). Duplicate treatments were harvested as two confluent T175 flasks (~20 million cells) for two control and two complex I-deficient patient cell lines, resulting in a total of 32 samples for RNA isolation.

### RNA sequencing

RNA was isolated using the Purelink RNA Mini Kit (Life Technologies). RNA was treated with DNAse and quality control was performed (OD260/280 = 1.8 ~ 2.2; OD260/230 ≥ 2.0; RIN ≥ 7.0; 28S: 18S > 1.0). Two 2 μg of RNA was analyzed using Illumina HiSeq2000 at least 10M clean reads per sample (BGI Genomics, Hong Kong).

### RNA-seq data analysis

Raw sequence data was filtered for reads that contain adaptors in their sequence (0.5 %) and with more than 50 % of low quality base calls (quality value < = 5, 1.1 % of total reads). 98.4 % or 10,270,698 of short reads passed these criteria. After filtering the reads were mapped to the reference using SOAP2 pipeline [41] allowing for 1 or 2 mismatched bases. Between 80 and 89 % of reads, depending on the measurement, were successfully mapped to the reference mRNAs, with 44 %–52 % uniquely to a single gene position. 18–24 % gene transcripts were covered across their full length (90–100 % of the transcript length) and 17–18 % of genes were covered for less than 10 % of their transcript length. Reads were subsequently mapped onto reference gene sequences to calculate RPKM (Reads Per Kb per Million mapped reads) [42]. This procedure resulted in expression values in at least one of the 32 samples for 19,426 genes. Of these, 13,700 genes were present in multiple experiments that allowed further analyses. The raw RPKM values were log-transformed and median-centered (median = 1.0). For analysis of the transcript levels of mtDNA-encoded genes we did not take into account non-protein coding genes, e.g., mitochondrially encoded 12S and 16S RNA (MT-RNR1, MT-RNR2) and tRNA leucine 1 (MT-TL1). Reproducibility of total mRNA measurements of the mtDNA encoded genes for the biological replicates was very high (R^2^ = 0.88, average difference between measurements 1.9 %; 0.1 %–12 %). The transcript levels of mitochondrial genes were normalized in the same way as nuclear genes.

### Co-expression calculations

For the complex I co-expression analysis we take into account all nuclear encoded subunits of the complex and its assembly factors NDUFAF1-4, TMEM126B [43], and ACAD9 [31]. Pearson correlations of expression were calculated for each of the 13,684 genes with every gene that encodes a complex I subunit or assembly factor. The correlation values are based on the expression measurements of the 32 samples. We then calculated for each gene the average co-expression with the extended complex I gene set (i.e., the genes mention above).

$$ \begin{array}{cc}\hfill c\left(g,S=\left[ NDUFA1, NDUFA2,\dots \right]\right)\hfill & \hfill =\underset{\begin{array}{c}\hfill g\hbox{'}\in S\hfill \\ {}\hfill \rho \left(e\left[g\right],e\left[g\hbox{'}\right]\right)\ge 0\hfill \end{array}}{avg}\rho \left(e\left[g\right],e\left[g\hbox{'}\right]\right)\hfill \end{array} $$

where *c*(*g,S*) is the co-expression of gene g with gene set *S*, where *S* is the extended set of complex I genes and assembly factors, *ρ* denotes the Pearson correlation, and e[g] is the expression vector in 32 conditions and cell types for gene g.

Similarly to expression patterns observed for all OXPHOS genes, complex I subunits and their assembly factors exhibit slightly divergent transcriptional programs. Additional file 2: Figure S6 reveals that complex I core subunits cluster together separately from assembly factors. The first (and largest) group is composed mostly of core subunits (from NDUFA11, top, to NDUFA6). Two smaller categories, with respectively seven and six subunits, additionally contain complex I assembly factors (NDUFAF1, NDUFAF5 in the second subclass; TMEM126B, NDUFAF2, NDUFAF4 in the third). Resveratrol and AICAR experiments appear to be most discriminative for the subgroup classification as the first subclass (subunits) shows low expression in resveratrol and AICAR in patients, and higher expression in AICAR-treated controls (Additional file 2: Figure S6). The smaller two subclasses of complex I genes do not exhibit such clear expression patterns. To account for the fact that subclasses fall under different transcriptional programs, we only considered Pearson correlations greater than 0. This increases the sensitivity of finding genes that fall under the transcriptional program of one of the subclasses (positive correlation), but not the other (negative correlation, which is not taken into account).

We selected a subset of 684 genes (representing the top 5 % of complex I co-expression scores) for downstream analyses. We determined this cutoff by three criteria: (i) it captures almost half (42 %) of the known OXPHOS subunits, (ii) it is close to a local peak in the frequency distribution of co-expression scores (Figs. 3 and Additional file 2: Figure S5), and (iii) the size of the resulting set of genes (684) is well suited for enrichment analysis, both on the level of functional classification and gene ontology as well as in terms of conserved transcription factor binding sites (see below).

### Conserved transcription factor binding site data

Data on conserved transcription factor binding sites (TFBS) in human were obtained from a comparative analysis of 29 genomes of placental mammals, such as primates, rodents and many farm animals [18]. In this study, transcription factor (TF) regulatory motifs were collected from the TRANSFAC [44] and Jaspar [45] databases, and several protein binding microarrays [46–48]. The presence of individual motif instances (putative TFBS) was predicted across the human genome based on conservation across the 29 mammals: for each motif match in human, the smallest phylogenetic subtree was calculated that contains the human motif and aligned motifs in other species [33]. TFBS were identified at a false discovery rate of 60 % and show reasonable agreement with experimentally measured ChIP-seq binding sites [18].

To identify putative regulators of a gene, we used conserved TFBS in promoter regions of genes, which were defined as 4 kilobase (kb) windows centered at all annotated transcription start sites of the gene (i.e. 2 kb upstream and 2 kb downstream of each transcription start site). This approach identifies instances for 361 regulatory motifs corresponding to 218 unique TFs (most TFs have multiple similar binding motifs) in the promoters of 16,298 genes, with a median of 7 (average 9.19) unique TFs per target gene.

### TFBS enrichment analysis

We used the Fisher’s exact test to calculate statistical over-representation of TFs regulating a gene set of interest compared to a background (e.g. all genes in the human genome, or all OXPHOS genes). Enrichment *P* values were corrected for testing multiple TFs (218 in total) using the Bonferroni or Benjamini-Hochberg false discovery procedures. TFs were judged to be significantly enriched at a significance level of 5 %.

## Availability of supporting data

The data sets supporting the results of this article are available in the GEO repository, GSE65634 http://www.ncbi.nlm.nih.gov/geo/query/acc.cgi?acc=GSE65634.

## Ethics

The study has been carried out in the Netherlands in accordance with the applicable rules concerning the review of Commissie Mensgebonden Onderzoek Regio Arnhem-Nijmegen. The board has approved this study and patients have provided written informed consent.

## Additional files

Additional file 1: Table S1. Overview of the structural subunits and assembly factors of OXPHOS complexes as used in this study. (XLSX 67 kb) Additional file 2: Figure S1. OXPHOS enriched cluster of genes. Figure S2. Mitochondrial OXPHOS genes respond differentially to treatments and assembly factors tend to express differently from nuclear genes encoding OXPHOS subunits (arranged by cell line). Figure S3. Total mitochondrial mRNA measured (RPKM) for mitochondrial protein coding genes. Figure S4. mRNA levels of mitochondrial genes encoding complex I subunits. Figure S5. Histogram (gray, left y-axis) and kernel density estimates (blue, right y-axis) of co-expression scores with known complex I genes. Figure S6. Dendrograms of the transcriptional response of genes encoding complex I subunits in 32 RNA-seq measurements. (DOCX 3004 kb) Additional file 3: Table S2. OXPHOS enriched cluster of genes. (XLSX 5387 kb) Additional file 4: Table S3. Top five percent complex I co-expressing genes. (XLSX 35 kb) Additional file 5: Table S4. DAVID gene functional classification results of the top five percent complex I co-expressing genes. (XLSX 27 kb) Additional file 6: Table S5. TF binding site enrichment analysis of promoter regions of the top five percent complex I co-expressing genes (full results). (XLSX 65 kb)

## Abbreviations

AICAR: 5-aminoimidazole-4-carboxamide ribonucleotide
AMPK: 5′ adenosine monophosphate-activated protein kinase
ATP: Adenosine triphosphate
ChIP: Chromatin immunoprecipitation
DMSO: Dimethylsulfoxide
ES: Enrichment score
mtDNA: Mitochondrial DNA
OXPHOS: Oxidative phosphorylation
RPKM: Reads per kilobase per million mapped reads
TF: Transcription factor
TFBS: Transcription factor binding site
