# Supplementary material for: Transcriptome analysis of complex I-deficient patients reveals distinct expression programs for subunits and assembly factors of the oxidative phosphorylation system
Source: BMC Genomics. 2015 Sep 15;16:691. doi: 10.1186/s12864-015-1883-8 (PMC4570683; doi:10.1186/s12864-015-1883-8)
Supplement: Additional file 2: Figure S1. — OXPHOS enriched cluster of genes. Figure S2. Mitochondrial OXPHOS genes respond differentially to treatments and assembly factors tend to express differently from nuclear genes encoding OXPHOS subunits (arranged by cell line). Figure S3. Total mitochondrial mRNA measured (RPKM) for mitochondrial protein coding genes. Figure S4. mRNA levels of mitochondrial genes encoding complex I subunits. Figure S5. Histogram (gray, left y-axis) and kernel density estimates (blue, right y-axis) of co-expression scores with known complex I genes. Figure S6. Dendrograms of the transcriptional response of genes encoding complex I subunits in 32 RNA-seq measurements. (DOCX 3004 kb) [file 12864_2015_1883_MOESM2_ESM.docx]

**
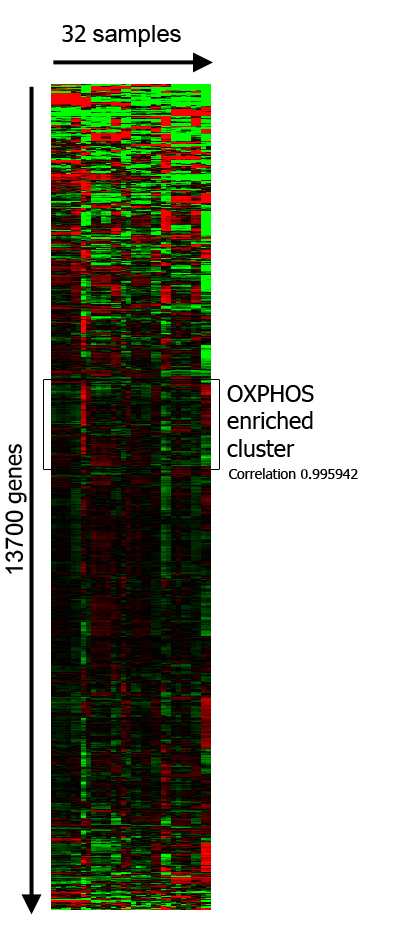
**

**Figure S1. OXPHOS enriched cluster of genes.** Heatmap representation of the expression of all detected genes in the 32 samples. One cluster of 1518 genes is significantly enriched for OXPHOS genes (Pearson correlation 0.995).


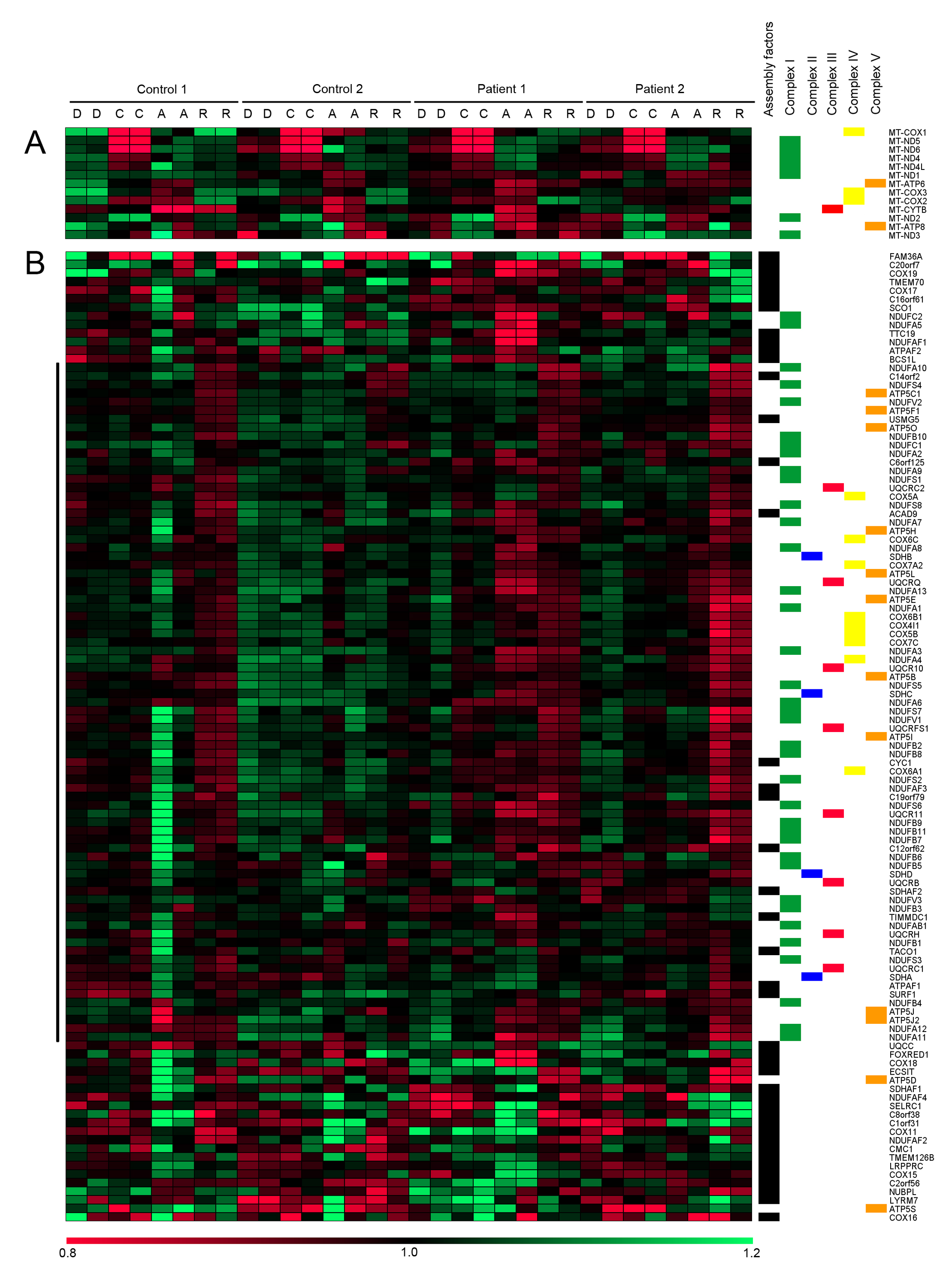
**Figure S2. Mitochondrial OXPHOS genes respond differentially to treatments and assembly factors tend to express differently from nuclear genes encoding OXPHOS subunits (arranged by cell line).** These are the data of figure 2 with samples arranged by cell line. In addition to the legend of figure 2: letters correspond to the treatment: D=DSMO, C=Chloramphenicol, A=AICAR, R=Resveratrol.

**Figure S3. Total mitochondrial mRNA measured (RPKM) for mitochondrial protein coding genes.** mRNA levels upon chloramphenicol (CAP) treatment are significantly higher compared to other conditions, denoted with a star (P-value 0.0003 or lower in pairwise comparisons, two-tailed paired T-test). Bars display RPKM measurements (mean ±SD) for all cell types and two biological replicates (total: 8 measurements).

**Figure S4. mRNA levels of mitochondrial genes encoding complex I subunits.** Each bar represents average RPKM measurements for all cell types and two biological replicates (total: 8 measurements).

**Figure S5. Histogram (gray, left y-axis) and kernel density estimates (blue, right y-axis) of co-expression scores with known complex I genes.** The red line marks a co-expression score of 0.54. The top 684 genes (from a total of 13,684) with higher scores than the 0.54 cutoff represent the top 5% of nuclear genes co-expressing with complex I. These genes are assessed in the functional classification and TF binding site enrichment analyses. Density estimates were calculated using a Gaussian kernel with a smoothing bandwidth of 0.005.


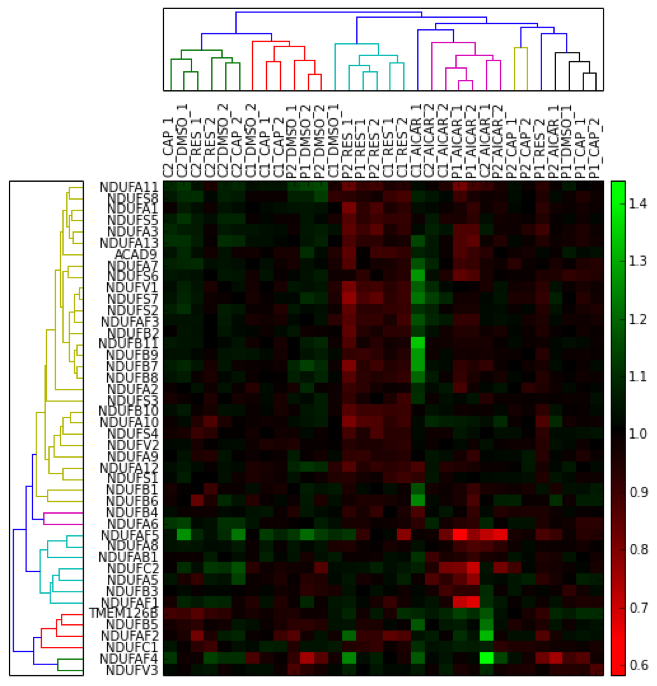


**Figure S6. Dendrograms of the transcriptional response of genes encoding complex I subunits in 32 RNA-seq measurements.** Complex I subunits and assembly factors NDUFAF1-4, TMEM126B and ACAD9 are included. Expression value 1.0 (black) denotes median log-expression of the gene (see Methods), with green denoting higher and red denoting lower expression levels.
